# Self-endangering: A qualitative study on psychological mechanisms underlying nurses’ burnout in long-term care

**DOI:** 10.1016/j.ijnss.2021.12.001

**Published:** 2021-12-15

**Authors:** Lara Luisa Eder, Bertolt Meyer

**Affiliations:** Department of Psychology, Wilhelm-Raabe-Str. 43, Chemnitz University of Technology, Chemnitz, Germany

**Keywords:** Altruism, Caregivers, Health personnel, Mental health, Nursing homes, Professional burnout, Social identification, Workload

## Abstract

**Objectives:**

To develop a more specific understanding of psychological mechanisms in the development of burnout in long-term care as a basis for potential new intervention strategies aiming at improving nurses’ mental health.

**Methods:**

Two qualitative studies with thematic analysis were conducted. In Study 1, we conducted eight group interviews with 110 nurses from May–July 2019 in the context of workshops at eight nursing homes in Germany. In Study 2, we supplemented these with semi-structured interviews with 14 executives at German nursing homes in December 2019.

**Results:**

The thematic analysis in Study 1 identified three main themes: causes of challenges, employees’ opportunities for change, and organisational opportunities for change. Thematic analysis in Study 2 identified three main themes: job motives, reasons for filling in for others, and employee self-care. Further, our results show that the need to stand in for colleagues, in particular, is one of the greatest challenges for geriatric caregivers. In dealing with these challenges we found that self-endangering behaviour—a diminished ability to say no when asked to fill in or to do work overtime—was an important antecedent of nurses’ burnout. Further, high levels of altruistic motivation and identification with the team or organisation were associated with self-endangering behaviour in the presence of adverse working conditions. Low levels of self-worth are a further risk factor for self-endangering.

**Conclusions:**

Our findings are at odds with some core tenets of classic models of job demands and burnout that construe motivation and identification as resources. Our results show the need of a holistic intervention program in nursing including individual coaching, team-based interventions and organisational development processes. Employees themselves should be sensitized to this issue and supported in the long term, and politicians should create structures that do not encourage this behaviour any further.

## What is known？

• Nurses are highly stressed and consequently show high burnout rates - these trends are increasing due to demographic change and emigration in the nursing professions.
• Theoretical models of demands and stresses highlight the importance of resources, e.g., self-efficacy or coping strategies.
• Little is known about specific psychological mechanism behind the resources that are crucial for improving the nurses’ mental health, e.g., team-based behaviour.
• Most of the recent studies used a quantitative approach and may not be holistic enough to be able to develop new intervention strategies.

## What is new？

• A qualitative analysis of psychological mechanisms in the development of burnout in long-term care was conducted under a specific perspective.
• The construct of self-endangering in nursing is considered as a mediating variable in the context of demands and the emergence of burnout.

## 1 Introduction

As in most countries, the elderly care system in Germany is facing major challenges due to the on-going demographic changes: As of 2019, the number of individuals requiring long-term care in Germany had doubled in the last 15 years to 3.8 million [1] and is predicted to rise to over 5 million individuals by 2050 [2].

As a result, the demand for caregivers will increase in the coming decades. At the same time, demographic changes will continue to increase the average age of caregivers. In addition, there is already an extreme shortage of skilled workers in nursing [3,4], resulting in a high density of work and resulting burdens for caregivers and nurses.

Given these issues, nurses are an important societal resource and are therefore particularly worth protecting. However, burnout is rampant among nurses [5]. Work overload, work-related stress, aggression at work, the intention to abandon the profession, and turnover intentions contribute to nurses’ burnout [6]. In addition, being single is associated with higher levels of nurses’ exhaustion, such that a lack of support complicates dealing with stress-related emotions in high demanding working situations [7]. Therefore, work design strategies and interventions for promoting nurses’ psychological well-being and protecting them against burnout are central for the sustainability of elderly care systems.

On the basis of central theoretical models of the antecedents of burnout—the job demands-resources (JD-R) model [8] and the conservation of resources theory (COR) [9], we already know that resources and demands interact with each other in their effect on employee mental health. According to the JD-R approach, job characteristics can be divided into job resources and job demands [10]. Job demands are defined as social, psychological, physical, or organisational aspects that require sustained physical or cognitive, or emotional effort or ability, and as a result, are associated with costs, e.g. emotionally demanding interactions [10]. Job resources refer to the physical, psychological, social, or organisational aspects of the job that are functional in achieving work goals and reducing job demands and the associated physiological and psychological costs or that stimulate personal growth, learning, and development [10]. Workplace commitment, a good working team, recognition from supervisor [11], stress management techniques, e.g., effective coping strategies [12] and work ethic feasibility [13] are helpful resources in dealing with stress. Even though we already know about important resources for dealing with stress from various studies, we still know little about their exact psychological impact mechanisms in nurses and their interaction with each other. However, from our point of view, this is indispensable for the development of targeted measures, such that nurses learn to reflect about and deal with their own resources, especially as long as politics does not create better care conditions.

For gaining more insights into the relation between nurses’ resources and burnout, we interviewed leaders and employees at eight nursing homes in Germany and analysed the transcripts using reflexive thematic analysis [14]. Reflexive thematic analysis is “a fully qualitative approach – with data collection and analysis techniques underpinned by a qualitative philosophy or paradigm approach” [14, p.6]. Given that we aim to develop theory rather than test it, this qualitative approach appears most suitable. It enables an active researcher role in the research process and allows a dynamic evaluation process such that new themes can emerge throughout the process [14], which for us is the decisive advantage over a quantitative approach when it comes to understanding (new) psychological phenomena in depth.

If we succeed in identifying psychological mechanisms behind important resources in dealing with stressful working situations in long-term care, we can, first, make an important contribution to empirical research on stress and strain in this target group and, second, develop new practical interventions. We hope that our results can also serve as starting points for holistic interventions in occupational health management in nursing homes.

### 1.1 Background

#### 1.1.1 Workload in nursing care

Shiftworking [15], heavy workloads [16], organisational problems such as time pressure [16,17], work overload due to understaffing [18], little control over work circumstances [19], and high levels of responsibility [20] characterize the working conditions in nursing homes. Further, we already know that nurses’ lower job satisfaction, lack of support, and feeling of poor leadership relate to higher levels of burnout and stress [21]. Nurses working in long-term care face additional challenges, as they provide more intensive care, and their clients often suffer from dementia [22] and challenge nurses with their behaviour [23]. Therefore, in sum, nurses are particularly vulnerable to burnout [24]. See the supplemental online file for more information on the special challenges in the residential elderly care system in Germany (Appendix A).

#### 1.1.2 Development of stress and burnout

For explaining the psychological processes underlying nurses’ exhaustion and burnout, two theories appear especially relevant, as they consider the interactions of demands and resources in the context of working: the JD-R model [8], and the conservation of resources theory [9].

According to specific resources, examples are social support [25], autonomy [26], and competence [27]. Further, clinical expertise, a good work-life balance, positive attitudes, managerial leadership, social support, and self-efficacy can prevent stress [13,28,29]. Moreover, personality patterns influence coping strategies [30], and active coping strategies mitigate the negative effect of high job demands [29]. Those resources can buffer several demands in the context of exhaustion [25,31,32] and therefore have great relevance for stress management in high-demand workings settings.

Moreover, COR theory proposes that demands diminish resources and that the (temporal) processes associated with resource losses cause emotional exhaustion [33]. According to COR theory, personal resources (e.g. self-efficacy) and social resources (e.g. close relationships) are central to individual’s identity, and individuals maintain psychological health by preserving these resources. The threat of resources, or an insufficient gain of resources after an investment or resource losses are the core antecedents of stress, burnout and exhaustion [9,34,35]. If we now assume, based on the difficult working conditions in long-term care that have persisted for years and the high burnout rates, that a loss of resources has already been ongoing for many years, it seems all the more important to understand how and why resources were lost and what it takes for rebuilding them.

Many interventions for health promotion have already been derived based on the JD-R and COR models. However, we can see from the increasing burdens reported in care [5,36,37] that the interventions are not sufficient. We posit that more knowledge is needed about psychological mechanisms and their interactions surrounding nurses’ psychological resources. Different levels of resources among team members may also affect individual team members’ health behaviour such that resource imbalances in the team can increase increases nurses’ exhaustion.

For example, COR theory posits that individuals with fewer resources are more likely to experience a further resource loss. However, the question arises as to which specific individual and team-based resources are needed in long-term care to compensate for the previous loss and to rebuild resources. Our efforts seek to answer this question.

### 1.2 The study

**Fig. 1:**
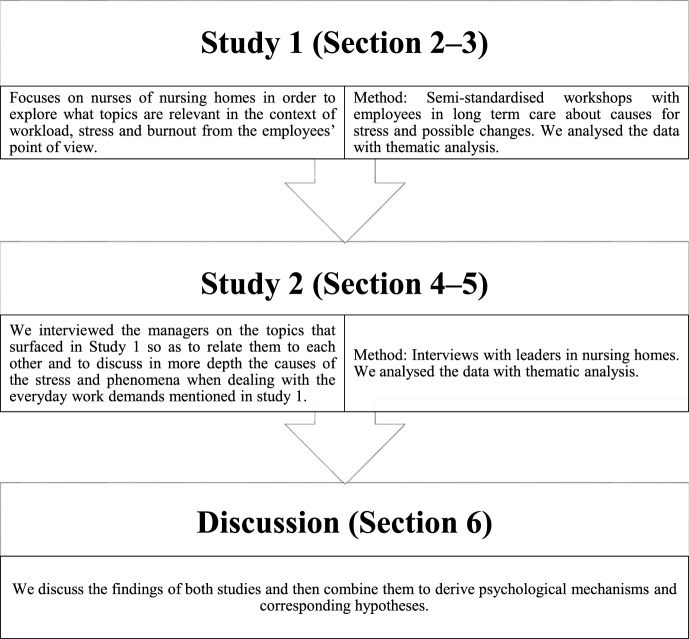
The present study’s procedure.

Previous research, especially today’s stress research, raises the question as to what other environmental or personal factors might protect nurses against job stressors [29]. We would go further to say that it is not only the question of further resources, but much more the specific psychological mechanism that is crucial for improving the nurses’ mental health. Although psychological factors are particularly important in dealing with stress, they are only examined in a few quantitative studies [[38], [39], [40], [41], [42]] and have not yet been integrated in terms of influences on individual’s and team-based behaviour. See Fig. 1 for a detailed description of the approach used in this study.

## 2 Methods of Study 1: employee workshops

### 2.1 Aims

The purpose of Study 1 was to 1) examine the causes of nurses’ burnout from the perspective of the employees themselves, and 2) elaborate what possible changes would help. We aimed to focus on specific people- and organisation-related causes and possible changes.

### 2.2 Design

We applied a qualitative study design using a reflexive thematic analysis approach. Thematic analysis is a data-driven method that identifies reoccurring themes or topics and relations among them in texts through inductively coding statements into sub-categories and categories [43]. In this way, the identified themes and patterns are strongly linked to the data themselves [44].

### 2.3 Sample/participants

We surveyed data from eight nursing homes located in North Rhine Westphalia in Germany that were participating in a large research project on health care. To represent the mixed cultures of nursing homes in Germany, they differed in the number of residents, sponsorship (private or state), and geographical area. We invited all the different professional groups in the nursing homes to participate through their area supervisors. The organisation leaders chose the participants of the workshops themselves, with a maximum of 25 people for one workshop. The sampling was influenced by low staff levels and the complexity of taking personnel out of their schedule. Therefore, a random or unbiased sampling was not possible, such that workshop participants consisted of nurses with a more constructive or critical approach towards their working conditions. We collected data in the summer of 2019.

The participants in the eight workshops conducted in the nursing homes were 110 of the nursing home employees in different functional areas (nurse specialists, nursing assistants, caregivers, social services employees, home economics, cook). Since the workshops were part of a wider research project, we were not allowed to define any study-specific inclusion or exclusion criteria. The only default we made was that employees’ participation was voluntary and that groups consisted of a mix of different functional and living areas. For reasons of anonymity and global project guidelines, we did not survey any demographic data. See supplemental online file for information about the health care project (Appendix B).

### 2.4 Data collection

Following the results of an employee survey (see Appendix B for detailed information about the covered scales), which was conducted in a previous analysis step in the health care project, the first author of this paper and another project partner selected two main topics individually for each nursing home, e.g. burnout, emotional labour, to be dealt with in the workshops at the beginning. Workshop participants could choose three further topics among the constructs collected in the survey, e.g. presenteeism, burnout, general health, work-privacy conflict. Participants then rated the importance of the potential topics by assigning points to each. The five topics that received the most points were then selected for the workshop. See supplemental online file for more information about the workshops and the detailed schedule (Appendix C).

### 2.5 Ethical considerations

Approval was obtained from the research ethics board of the university. At the beginning of the entire research project, all nursing home leaders were informed comprehensively about the project, its contents, methods and objectives in one-on-one meetings and steering committees. In addition, employees were informed through staff meetings and written notices. All participations provided informed consent. The employees all participated in the workshops voluntarily and during their working hours. The objectives and content of the questions were made clear several times at each stage. The workshops were anonymous, so no personal data was collected. Furthermore, no managers took part in the workshops, in order to create a confidential and protected atmosphere for the employees.

### 2.6 Data analysis

The employees’ individual answers to the questions on the causes and the respective change possibilities in the workshops were recorded in keywords in columns on flipcharts. We subsequently transferred these keywords into MAXQDA for analysing the data using thematic analysis [43]. We did not return transcripts to participants. The existing cooperation with the organisation helped us interpret the data, as Braun et al. (2018) [14] also mentioned that the aim of thematic analysis is not to exactly summarize the data and minimize the researcher’s subjectivity. The aim is much more to provide a coherent interpretation grounding in the data by the researcher playing an active role [14].

Given the aim of the study, we analysed the results of the questions “What are the causes of [topic] (for example, high burnout symptoms, high rates of presentism), what must happen for the condition to improve, what can I contribute to that, and what can the institution contribute to that,” because we wanted to focus on causes and possible changes. We followed the six phases of thematic analysis [43] for the evaluation. After transcribing the data, we reread the issues referred to the workshops. As thematic analysis is a data-driven method [43], we inductively coded the participants’ statements, which consisted of a few words or short sentences, and initial discursive themes. That means that the identified themes and patterns are strongly linked to the data themselves [44]. The themes were grouped and then reviewed for overarching major themes, variability, and consistency. These themes were interpreted through a process of reading and rereading, as well as reference to relevant literature and consultation with colleagues. We then formed main codes and subcodes. Our implicit knowledge on the organisation and structures helped us to move beyond the surface or obvious content [14]. During the coding process, we grouped themes or separated them if a thematic differentiation appeared necessary, so we always paid attention to identifying new codes.

The whole category system evolved dynamically throughout the coding process, and all eight workshops were coded. We stopped coding when all transcripts were assigned to an appropriate category and the description of the coding system was complete. When the coding process was completed, we checked and rechecked the themes and discussed them with two colleagues who work in the psychological sector. If a statement was not appropriate to a theme, we discussed that and, if applicable, built a new theme. We then discussed the themes’ areas of overlap and the identified relations in-between, which we summarized for visualizing in MAXMAPS.

After that, based on the frequency distribution of the topics, we identified the most frequent topics from the employees’ perspective.

### 2.7 Rigour

To improve the rigour of thematic analysis, we used the MAXQDA computer software [45]. Further, we tried to support the validity of data using verbatim quotes [46]. We ensured trustworthiness by following the Lincoln and Guba (1985) [47] criteria during our research process. Specifically, we ensured credibility through the familiarity between the main researcher and the nurses: By interacting with the nursing homes and their employees during the health care project, participants were already familiar with the first author for eight months when the workshops started, and a baseline level of trust had emerged. To ensure confirmability and dependability, we conducted several peer debriefings and four expert exchanges about the code assignments and theme building during the coding process. All experts had a psychological background, such that three had at least a master’s degree in psychology and one was in advanced master’s studies with additional practical work experience in the field of training. Two of the other three were researchers in organizational psychology and one had a clinical psychology background working in a hospital. All experts were familiar with the nurses' working situations but were not part of the practical project activities in the nursing homes. Additionally, we implemented the peer debriefings with our health care project partners from occupational health management. In sum, we were able to bring together a good mix of scientific and practical experience. To increase transferability, we have prepared a detailed description of the contextual factors of the health care project, the nursing homes and the samples. See the supplemental online file (Appendix B).

## 3 Results of Study 1

The thematic analysis identified three main themes: causes of challenges, employees’ opportunities for change, and organisational opportunities for change. Table 1 gives the central themes and subthemes, definitions, examples, and frequencies.

**Table 1:** Study 1: Themes and subthemes.

Themes and subthemes			Definition	Example	Frequency
**Causes of challenges** (190 codes)	Structural working conditions	Work structure	Causes that lie in the area of working conditions and relate to work design features such as information sharing/exchange, lack of time, responsibilities, task design/diversity and equipment features.	*“Too many tasks in too short time for care assistants.”* (NH 3)	52
		Working hours	Causes that lie in the area of working conditions and relate to working hours, shift work and filling in for others.	*“Many working days are planned one after the other.”* (NH 5)	20
		Personnel structures	Causes which relate to staffing, qualifications and absenteeism in care.	*“We are constantly understaffed.”* (NH 5)	13
	Employee’s psychological characteristics	Missing self-care	Causes of negative stress due to lack of self-care behaviour/awareness and associated cognitions of employees.	*“One does not think of oneself.”* (NH 8)	17
		Attitudes and values	Causes of negative stress caused by concrete values and attitudes towards work.	*“When you are ill, you feel guilty towards colleagues and residents.”* (NH 5)	14
	Team culture and climate		Causes of negative stress caused by the interaction in the teams and relating to behaviour and attitudes.	*“You feel guilty when you bring something up.”* (NH 1)	35
	Leadership behaviour		Causes caused by leadership behaviour.	*“It is often not accepted if you cannot fill in - you are supposed to postpone your private appointments.”* (NH 5)	19
**Employees**’ **opportunities for change** (117 codes)	Self-care	Distancing oneself from something	Specific aspects of self-care behaviours that relate to being able to say no and to consciously setting boundaries for employees. Some mention that this capability depends on the occupational group and others on individual characteristics.	*“Learning to say ‘no’ and also think of yourself.”* (NH 5)	20
		Separation of work and personal life	Specific aspects of self-care behaviours that relate to balancing and separating work and private life.	*“Actively using one’s own free time for regeneration, knowing what is good for you.”* (NH 7)	11
		Self-reflection	Specific aspects of self-care behaviours that relate to engaging with and reflecting on oneself as a person.	*“Understanding and perception of own needs and deficits.”* (NH 4)	9
	Behaviour in the team		Possibilities for change that relate to behaviour or attitudes in the team/among colleagues.	*“Openly state in the team that ‘it’s all too much for you’ and that you are therefore irritated.”* (NH 3)	31
**Organisational opportunities for change** (196 codes)	Leadership	An understanding attitude	The employees want a supporting and understanding management and that they take their concerns seriously.	*“See what has been achieved despite poor staffing (don’t take it for granted.”* (NH 7)	14
		Behaviour	Leaders should take responsibility and fulfil their role model function.	*“Management must bear responsibility for agreements.”* (NH 8)	10
		Feedback and recognition	The employees want praise and recognition to be lived and felt in the facility.	*“Appreciation from all levels for all levels.”* (NH 7)	10
	Work organisation	Personnel management	From the employees' point of view, there is a need for more staff and better care keys.	*“We need more staff.”* (NH 6)	20
		Times for exchange	Employees want more times for exchanges for all professional groups within the nursing homes.	*“During handovers, the assistants should also be present at the start of the shift.”* (NH 1)	19
		Organisation in case of illness	The same people should not fill in permanently and others should also be asked or a pool of so called stand-ins introduced.	*“Do not call when free/holiday/sick and ask if you can fill in.”* (NH 3)	18

### 3.1 Theme 1: causes of challenges

This category contained the antecedents and causes of job-related challenges that the nurses identified. These included especially structural working conditions (e.g. working hours, personnel structures) and employee’s psychological characteristics such as missing self-care or attitudes. With 98 mentions, participants referred to the structural working conditions the most frequently, followed by employee characteristics such as missing self-care and employees’ attitudes and values with 38 mentions, team culture and climate with 35 mentions, and leadership behaviour with 19 mentions (see Table 1).

Employee’s psychological characteristics, team culture and climate, and leadership behaviour were the vaguest themes, so we present some examples. Employees’ characteristics as a cause of challenges included missing self-care, missing self-efficacy, and employees’ attitudes and values. Missing self-care means not being able to switch off after work, being too tired to do something in your free time, the thought to still be able to do more, meaning that one does not think of oneself and that employees’ would rather save the other persons weekend than their own.

*“I could do more.”* (NH 1)

*“You put other people before yourself.”*(NH 8)

Employees’ attitudes and values mean especially a strong sense of responsibility and duty and having a bad conscience towards colleagues and residents.

*“I would rather save the other person’s weekend than my own.”* (NH 6)

*“I have feelings of guilt towards colleagues and residents.”*(NH 5)

The team culture and climate included aspects such as remaining silent for fear of consequences, lack of confidence, hierarchical differences between the professional groups, lack of cohesion among staff, ignoring each other, or talking about someone behind their back:

*“Colleagues relying too much on others.”* (NH 3)

*“You don’t want to stab others in the back.”* (NH 6)

The leaderships aspect included a lack of praise and appreciation, pressure, and a lack of acceptance for employees who do not fill in for others.

### 3.2 Theme 2: employees’ opportunities for change

This category contained opportunities for change that the nurses identified. The most frequent topics were self-care (see Table 1, mentioned 61 times) and behaviour in the team (see Table 1, named 31 times).

*“I could openly say to the team, ‘it’s all too much for me’.”* (NH 3)

Self-care was in turn divided into five further sub-categories: self-efficacy, self-reflection, consideration of the state of health, separation of work and personal life, and the most important topic from the employees’ view: distancing oneself from something. Distancing oneself from something means, on the one hand, being able to set physical and especially mental limits regarding residents, the leader, or colleagues and, on the other hand, being able to say no when the leader or colleagues ask you to step in for another employee in the event of absenteeism.

*“I could learn to say ‘no’ and think of myself.”* (NH 5)

*“I could put ‘the brakes on myself’ more often.”* (NH 1)

*“Your own awareness of being able to do something and wanting to do something.”* (NH 7)

### 3.3 Theme 3: organisational opportunities for change

This category contained the organisational opportunities for change that the nurses identified. The most frequently mentioned topics were work organisation (named 99 times, see Table 1) and leadership (named 40 times, see Table 1). Within work organisation, the most important themes from the employees’ view were personnel management, times for exchange, and organisation in case of illness. Within the theme leadership, the employees pointed out that they want an understanding attitude and recognition from the management.

*“They could accept a ‘No’ without making me feel guilty.”* (NH 3)

*“They could give me the feeling that it is allowed to say ‘No’ (e.g. when they ask me to fill in for others).”* (NH 1)

## 4 Methods of Study 2: semi-structured interviews with leaders

### 4.1 Aims & design

The aims of Study 2 were to 1) examine the causes of nurses’ burnout from the leadership and employee perspective, and 2) elaborate potential changes or interventions that might help to mitigate burnout. We aimed to focus on specific people- and organisation-related causes and possible changes. We used the same approach as mentioned in Study 1.

### 4.2 Sample and participants

At each of the participating nursing homes, we asked for two or three executives to participate in the interviews during the health care project process. None of them knew about the aim of this study, and this research project was independent from the health care project process or aims. We collected data in December 2019.

Fourteen executives in different units at six of the eight nursing homes took part in the interviews: 12 women and two men aged 36–58 years (*Mean* = 45.86, *SD* = 7.36). The mean years of affiliation with their organisation ranged from 1 year to 3 months to 33 years (*Mean* = 11.23, *SD* = 10.68).

The participants were divided into their functional areas at the nursing homes: four directors of the facilities, two living area managers, two home economics managers, two nursing service managers, and two social service managers.

### 4.3 Data collection

We used semi-structured interviews to ensure that the executives were guided but not limited in their answers. We gave them no restrictions in their explanations, and we added exploratory questions to gain a deeper perspective on what was being said.

Before the interviews, we sent the information and declaration of consent form via email to the voluntary participants. Three of the 14 interviews were conducted via face-to-face conversations and 11 via telephone. Before starting, we asked for consent for the interview to be audio recorded. Interviews lasted between 8 and 33 min, with an average interview time of 16 min 40 s.

A guideline for the interviews was prepared in advance that included all topics relevant to the survey and their possible interrelationships. Conducting semi-structured interviews allows the interviewer, for example, to adjust the order of questions and to respond to the answers of the individual interview participants [48].

Although we analysed the results of the workshops in study one in a standardized way in MAXQDA after carrying out the interviews, we already knew a lot about the employees’ most important topics and correlations from the workshops. The identification of the topics for the semi-standardized interviews with the executives was therefore based on the answers of the employees in the workshops. For the interview questions, see the supplemental online file (Appendix D).

### 4.4 Ethical considerations

Approval was obtained from the research ethics board of the university. In addition, the consent of all facility managers and the corresponding works councils on site was obtained throughout the research project. All interviewees were informed in advance in writing about the procedure and the contents and asked for written consent to participate. In addition, another verbal explanation was given on the day of the interview, and only after the participants had given their consent were the interviews recorded by telephone for later evaluation. Demographic data were requested prior to recording, so that they were fully anonymised. We have no further ethical concerns regarding the study, as we did not deceive or manipulate the target group in the course of the research.

### 4.5 Data analysis

We fully transcribed the interviews into MAXQDA according to Dresing, Pehl and Schmieder’s (2018) [49] semantic transcription rules for analysing the data using thematic analysis [43]. We did not return transcripts to participants. Due to the fact that the first author conducted the interviews and afterward the transcription herself, all uncertainties and supposed misunderstandings were clarified directly in the interviews. Nevertheless, we contacted the leaders via telephone in case of uncertainties or questions. After transcribing the interviews, the first author of this paper reread the contents several times. The first author carried out the interviews with the leaders, so she was already familiar with the content. We coded the interview statements in an inductive-deductive way. Based on the interview questions we built the upper categories in a deductive way and then following the sub-categories in an inductive way directly on the interview material. During the evaluation phase, the category system grew from interview to interview. The coding process was the same as described in Study 1 above.

As described in Study 1, we grouped themes or separated them if a thematic differentiation appeared necessary, so we always paid attention to identifying new codes. We stopped coding when all interviews were coded and all aspects were assigned to an appropriate category.

The whole category system evolved dynamically throughout the coding process, and all relevant text passages from the 14 interviews were coded. When the coding process was completed, we checked and rechecked the themes and discussed the patterns and relations with two colleagues who work in the psychological sector. If a statement was not appropriate to a theme, we discussed that and, if applicable, built a new theme or renamed themes. After we put the themes in relation to each other, we summarized the results for visualizing in MAXMAPS.

Based on the frequency distribution of the topics, we then identified the most frequent topics from the leaders’ perspective.

## 5 Results of Study 2

Thematic analysis identified three main themes: job motives, reasons for filling in for others, and employee self-care (Table 2).

**Table 2:** *Study 2: Themes and subthemes*.

Themes and subthemes			Definition		Example	Frequency
**Job motives** (44 codes)	General conditions		Nursing staff are motivated to exercise their profession due to structural conditions.		*“The opportunities for advancement are now also mentioned. So you really have opportunities to educate yourself, to further educate yourself and yes, to make a career.”* (P 13)	8
	Social motivation	Want to help others	Care workers are motivated to want to help other people.		*“Let’s put it this way, I’ll try to put it another way. It*’*s more like that helper syndrome that’s going on. Which may not be so obvious to you either. And says yes, I’m totally interested in helping people somehow from the nursing point of view, because they are so limited.”* (P 2)	13
		For what comes back from the inhabitants	Nursing staff work for what they get back from the facility's residents.		*“So, in all these years what I’ve noticed is what comes back from the resident. You get a lot in return. And I think it’s one of those, of course, there are also some who just go to work because of the money, but most of them are there because a lot comes back. In gratitude, in appreciation (laughs) and that really is the most. Are there maybe 1–2 inhabitants where it is different, but mostly it is so and that was also with me. So, to have that feeling, they need you, they are grateful, they just love you. When I come back from vacation, thank God they are back. And that’s the beauty of it. The residents think of you and think one more week then she comes back. And that’s just what comes back from some.”* (P 4)	2
		Having relationships	Nursing staff work in the care sector to interact with the residents or other colleagues.		*“(…) is that they like to work with people, that they like to work for people, that they want to work with people because they want to cultivate relationships, are communicative, so they like to be communicative, so they like to have conversations.”* (P 13)	14
**Reasons for filling in for others** (133 codes)	General conditions for coverage		Framework conditions for loss compensation in inpatient care for the elderly include compensation by internal or external employees, the importance of the issue for the employees, the responsibilities and regulations for this loss compensation, and dynamics that arise in the course of loss compensation.		*“First in the team itself. I must honestly admit that sometimes I only get the information that a colleague or a colleague is ill, but that is often regulated directly by the management of the residential area or the coordinating service, who ask the ( …) colleagues to step in.”* (P 13)	15
	Duty of managers to provide for the welfare of their staff		Managers in the care sector have a duty of care towards their team and must keep a close eye on which employees tend to always say yes and step in to protect them and balance the team's step-in behaviour.		*“(…) that as a manager you also have to protect some people who you don’t call because you then know exactly that they will always fill in. That is the art of doing it more or less fairly.”* (P 9)	11
	Employee substitution behaviour		Characteristics of nursing staff's entry behaviour.		*“Yes. So, there are always those who come very often or always, and there are those who, wherever they feel like that, you have to beg or they don’t come at all.”* (P 2)	20
	Differences between employees who fill in for each other and those who do not	Identification with the residents	Differences in identification with the residents, which justify the (non-) substitution of employees.		*“Yes, and then there are simply employees who see the necessity, because the residents must be cared for.”* (P 6)	3
		Intrinsic motivation	Differences in the intrinsic motivation of the employees, which are the reasons for the (non-) substitution of employees.		*“There are employees who have a general willingness, who like to do this from the heart.”* (P 8)	5
		Team spirit	Differences in attitude and understanding of his or her team/colleagues, which justify the (non-)replacement of employees.		*“Yeah, I would just say they identify with the team, the people who fill in. So, and the others, they don't really care. Yes, they don’t identify with the living area, not with the team, they just do their job and only do what is necessary, what goes beyond that.”* (P 12)	12
		Personal characteristics	Differences in the personal attitudes and personality traits of employees, which justify the (non-) substitution of employees.		*“Oh, helper syndrome probably (laughs). There are people who just can’t help themselves.”* (P 4)	10
		Structural conditions	Differences in the framework conditions in inpatient nursing care that favour or make it more difficult for staff to fill in.		*“Well, I do have employees who I know will find it difficult to fill in because of their family situation or their private situation.”* (P 14)	9
	Employees’ approach to covering for absent employees	Alternatives	In the event of necessary compensation for absenteeism, the employees are not contacted/called in their free time under various circumstances and the loss is compensated for by an on-call standby/background service, the existing staffing with local employees, partial services or the replacement of the manager himself.		*“Well, I say, if the on-call service, as I call it now, has already been activated, then there are possibilities to take a look inside again, maybe another living area is better occupied and can be helped out completely or by the hour. And you just have to look at the fact that we have already reached the minimum cast. Well, that’s certainly an issue for now.”* (P 11)	17
		Call employees in their free time	**Part-time employees**	Part-time employees are often asked (first of all) whether they can fill in when a loss has to be compensated.	*“These are of course often the part-time employees. Someone who has a day off full-time will be called less.”* (P 5)	4
			**Favourites**	The managers in the inpatient nursing care sector do not follow a number-based step-in principle in their approach to compensation for loss of earnings. This means that they often ask those employees whom they know will say yes.	*“So, the feedback that I got for example from the night shift from earlier times is that, because I also know that one or the other colleague does not agree at all and that you choose your fingers sore and ( …) well, I think you have your favourites, where you just know that you can call, support them and they will jump in.”* (P 13)	7
			**Principle of fairness**	The managers in the inpatient nursing care sector follow a fair step-in principle in their approach to compensation for lost time. This means that they take into account working hours, overtime or past rhythms of filling in for their decisions about which employees to ask.	*“One calls, so first I call everywhere where I probably get to hear no and then I reach out to those who usually say yes. But I still try to call the others first, because they have to fill in the same way and not always the same ones.”* (P 4)	8
**Employees**’ **self-care** (78 codes)	Management’s responsibility		The management responsibility describes the duty of care and the responsibility of managers to look after the health of their employees and to act as a role model.		*“But we actually try to work as leaders to say, take care of yourself and it is also ok to take a 5-min break, or to take a deep breath, to get out of the situation, especially if it is a very emotionally and psychologically stressful situation, because afterward you can simply continue working better than not taking a break.”* (P 7)	6
	Attitude		The different attitudes of people who take good or less good care of themselves.		*“I think in all areas you have people who say it doesn’t matter, the work has to be done, I’m going all the way.”* (P 1)	7
	Meaningfulness		The importance of self-care in inpatient elderly care describes the importance of this topic from the perspective of managers.		*“Well, it matters a great deal. So that really plays a role, that is, to understand what is meant by self-care and to really take that seriously, I think that is, from my own experience I must say, incredibly important.”* (P 9)	6
	Break culture		The break culture in the elderly care describes behavioural patterns with regard to taking and organising breaks, the acceptance of breaks and differences between smokers and non-smokers.		*“I mean the smokers, they always go out for a smoke, take a break. But the non-smokers, it is more difficult to take a break then. Unless you want to go outside for some fresh air. But I think smokers find it easier.(…) Right, because you have no reason to go out. ( …) No apparent at this moment, no inner urge.”* (P 8)	12
	Self-care capabilities		The theme includes all aspects relating to the importance, awareness, work-life balance or the development of self-care behaviour among employees. This category also includes factors influencing self-care behaviour.		*“( …) there are especially among the skilled workers employees, who sometimes have the tendency to go beyond their limits and not so for themselves, now it’s time to go home, now I need my time off.”* (P 6)	18
	Ability to distance oneself from something	Ability to say no	The category describes the ability to differentiate oneself in the form of saying no when asked to step in.		*“(…) and all those who have this background working with people in this way often have a problem saying no, because they tend to put others in the foreground rather than their own needs.”* (P 7)	7
		Depending on occupational group	The category contains all aspects of the topic “ability to differentiate” from the point of view of the interviewees that are dependent on or related to the occupational group or activity assignment in the institution.		*“On the whole, nursing is by and large the professional group that finds it very difficult to say no, and in the area of care we really have a similar situation.”* (P 6)	12
		Depending on individuals	The category contains all aspects of the topic delimitation ability from the point of view of the interviewees which are dependent or related to the person or which are individual.		*“I don’t think that is necessarily dependent on the professional group, no I don’t think so. I think this is a very individual topic.”* (P 11)	10

### 5.1 Theme 1: job motives

This category contained the different job motives that had motivated nurses to choose their job. These included social motivation and general conditions that the profession entails.

The general conditions in nursing, such as earning money, being able to make a career, or to have a secure job were a less common reason for working in a nursing home (named 8 times, see Table 2). The most common motive for people working in nursing homes in the view of leaders was social motivation (named 29 times, see Table 2). We differentiated the social motivation into three sub codes and found that helping and having relationships with others were the strongest motivators for employees working in the context of nursing. Having relationships meant relationships with the residents and developing relationships with your colleagues.

*“Employees go into a deep level with them.”* (P7)

*“Have a feeling of being needed.”* (P4)

*“Feel that the residents, they just love you.”* (P4)

*“Well, it is such a (…) good network that you build up, besides the family structure or acquaintances and friendships that you cultivate. You must have colleagues there as well. I think you profit from it very, very, very much. Teamwork, teamwork is needed.”* (P13)

In the context of helping, some of the respondents also mentioned what is so called the helping syndrome, meaning that employees in nursing homes are often over-motivated to help others and that this can sometimes be pathological.

*“It’s more like that helper syndrome that’s going on, which may not be so obvious to you either. And says yes, I’m totally interested in helping people somehow from the nursing point of view, because they are so limited. (…) It may be pathological or not, it doesn’t matter. (…) Yes, if no peace comes in there. If you feel like you are always driven by love for people and you want to help other people all the time and are NEVER free of that, then I think it’s pathological, so it goes along with it to such an extent that I think it’s really pathological.”* (P2)

### 5.2 Theme 2: reasons for filling in for others

This category contained employees’ approach in the event of necessary coverage for absent employees, the general conditions for coverage, differences between employees who fill in for others and those who do not, employee substitution behaviour, and duty of managers to provide for the welfare of their staff.

#### 5.2.1 Employees’ approach to covering for absent employees

In the event of necessary coverage for absent employees, the leaders reported that they pursue two approaches. They can call in employees in their free time to cover for the absenteeism, or they can try to cover for absent employees by implementing an on-call standby service system (this was implemented in only one of the participating nursing homes and only for care assistants), by covering via the existing staff on site, by sharing the working time for the present staff, or by leaders stepping in themselves.

When none of these alternatives are possible, the leaders usually must make use of employees who are on leave (named 19 times, see Table 2). When we asked which employees the leaders call first, they mentioned three things that they do. First, they call part-time employees.

*“There are people who often fill in, these are our part-time employees. Because you call them first because they are more often out in the open.”* (P5)

Second, they call employees that they know will agree to come in.

*“You call actually, you call those who you know they will come in, you call them first.”* (P10)

Third, they take overtime and replacement rhythm into account when deciding who to ask first:

*“Then it was actually fairness, because I always think fairness is above everything. The one who has the least overtime, he is asked first, if he can’t, the next one is asked. So, we looked at the numbers or the rhythm, how often did one person step in and how often did the other. So that everyone feels treated equally, and then you just go through, he was already there, has the least overtime, so the next one comes in.”* (P2)

#### 5.2.2 Employee substitution behaviour

From the leaders’ point of view, all reported that they have employees who always stand in for their colleagues when coverage was needed and agree when they are asked, and all have employees who never fill in for their colleagues and everybody knows that they will not agree when they are asked.

*“There are people who would always fill in, even if they have worked—I would say—maybe even too much. They would fill in anyway. And others always have other appointments.”* (P11)

#### 5.2.3 Differences between employees who fill in for each other and those who do not

The differences between employees who fill in and those who do not could be divided into five subthemes: identification with the residents, intrinsic motivation, team spirit, personal characteristics, and structural conditions.

One of the two most frequently mentioned differences between employees was in team spirit. Team spirit means that employees who fill in for each other do not let their colleagues down, they stick together and they do not want to abandon their team.

*“They want to be a good colleague.”* (P8)

*“The attitude could be, I won't let the team down so that could be an attitude. And some people don’t care, so if you don't step up then. Maybe they don’t have such a strong bond to the team or the institution, so maybe the identification is different, I could at least imagine.”* (P11)

The second most frequently mentioned difference between employees was in personal characteristics, which means personality traits and attitudes. It was mentioned that employees who often or always fill in for others feel extremely responsible, are particularly selfless and helpful, put their private lives on hold, and have a pronounced helper syndrome.

### 5.3 Theme 3: employees’ self-care

This category contained the management’s responsibility for employees’ health care and self-care, the general attitude to self-care, the meaningfulness of self-care, break culture, self-care capabilities, and the ability to distance oneself from something (Table 2).

#### 5.3.1 Self-care capabilities

All the participating leaders agreed that the majority of the employees have only limited abilities to look after themselves (named 18 times, see Table 2).

*“Yes, exactly, so how should I say, those who are especially connected to the work or the wearer, I think they don’t really take care of themselves, including me (laughs).”* (P10)

They described was a lack of awareness of self-care on the part of employees and stated that many do not know what they can do. It was also argued that one reason for the low self-care capabilities is the helping syndrome and that people who work in the care context often tend to be selfless.

*“There are especially among the skilled workers’ employees who sometimes tend to go beyond their limits and do not say to themselves, now it’s time to go home, now I need my time off.”* (P6)

Further, some executives saw an age effect. They reported that older employees often react selflessly and should do more self-care than they actually do.

*“I believe I would also like to make this clear to the older employees, who have gone through the old school, I say it now times through the old school, still have such old pictures in their heads. Without me, it does not work and I must somehow go on until ( …) until it is no longer possible, so they really react very selflessly. And so, with the younger employees who come, who, for example, want to combine family and career, but who then also set a few conditions, this sometimes meets with irritation between the generations. You have to do justice to both. For those who are still socialized differently and have gone through the old school of nursing care for the elderly and for those younger employees who, I believe, have a completely different approach to the work-life balance. So, I think you have to create a good understanding of each other. The younger ones I think they already have a different one, so I hope they have a different view of the working conditions and (…) yes, I think they are always the older employees, by older I mean also those who are my age, we are the ones who are perhaps still a bit of the old breed or I would say we got to know them differently. So, the older employees really have to, I think sometimes they have to do a bit more for themselves.”* (P13)

#### 5.3.2 Ability to distance oneself from something

##### 5.3.2.1 Ability to say no

The employees’ ability to say no when they are asked to fill in for their colleagues or do some extra work was one of the main themes in the interviews. All leaders mentioned that saying no is accepted and necessary for a good self-care in the nursing homes. Conversely, this ability is only very slightly developed among the employees.

*“(…) and all those who have this background working with people in this way often have a problem saying no, because they tend to put others in the foreground rather than their own needs.”* (P7)

##### 5.3.2.2 Depending on occupational group or individual characteristics

Executives reported that employees’ ability to say no depends on the professional activities and thereby on the occupational group in the nursing homes. Especially for the nursing stuff, it is usually difficult to say no, whereas for example for the social services, it is often easier because more flexible in terms of timing. To put it more generally, some processes in the nursing home encourage the employees’ ability to distance themselves from something because they can be rescheduled or cancelled. Further, it was mentioned that the social services staff sometimes know more about themselves or self-care strategies because they learned this during their education and training.

*“My experience is that sometimes nursing staff can not do that. Because they really do have the attitude that they have to help people and on the other hand they have to have this attitude, so that they can work well or are good and what we want for our residents, and that really is a greater challenge in the nursing field than in some other occupational groups in the home.”* (P9)

In contrast to the executives’ view concerning factors influencing the capability to distance oneself from something as described above, some leaders also mentioned that this capability depends on individual aspects.

*“I would say first of all that it also strongly depends on the employee’s personality.”* (P13)

### 5.4 Self-endangering in nursing

Our results show a vicious circle of high stress and little self-care causing health problems and absenteeism. This absenteeism in turn requires flexibility and coverage by others, which leads to more stress for the nursing staff. With regard to the boundary conditions, our study participants see the greatest potential for change in their own self-care and in the behaviour within the team.

In our two qualitative studies, we investigated nurses’ challenges and associated boundary conditions in long-term care in order to examine the psychological mechanisms behind individuals’ resources or even lack of resources.

Overall, the thematic analyses led to the main topic of accepting extra tasks or filling in for colleagues in cases of illness or absence, which usually results in overtime, long and frequent work shifts in a row, presenteeism, and lack of recovery. Our results indicate that employees' interpersonal differences in team identification as well as in the personal characteristics determine the way that nurses in long-term care deal with and react to these special demands. One of the most important themes for employees and executives seems to be the ability to say no and the associated general ability to set oneself apart from colleagues and long-term care residents. Participants mentioned that they “find it important for self-care to say no and to say no, but unfortunately, I can’t now.” A lot of participants believed that “there are many who have a particular problem saying no.” Overall, a large number of statements reflected self-sacrificing behaviour among nursing staff. In summary, on the basis of our qualitative results, we construe self-endangering as a central mediator between nursing-specific characteristics and burnout. Basically, based on our findings, we assume that two facets of self-endangering at work can be classified: behaviour and behavioural tendencies and cognitions. We define self-endangering behaviour as a diminished ability to say no when asked to fill in or to do overtime. We define self-endangering cognitions as the nurses’ own inner beliefs with regard to having a moral obligation to fill in for colleagues at the expense of their own health.

As we see the introduction of the construct of self-endangering in the context of nursing as a central contribution, we will focus on this aspect and its connections to the mentioned individuals’ resources and characteristics of nurses in the discussion.

## 6 Discussion

### 6.1 Self-endangering in nursing

To our knowledge, there has been only little research on self-sacrificing behaviour in the professional context, but we propose that the concept of self-endangering work behaviour is close to what we found in the context of self-sacrificing behaviour in nursing. Self-endangering behaviour is defined as “actions that aim to deal with work-related demands but simultaneously increase the likelihood of health problems and impede necessary recovery from work-related stress” [50, p.28]. These are, for example, behaviours that are internalized but have a potential health risk, such as extending work time, reducing recovery time, intensifying work, and presenteeism [51]. Self-endangering also includes goal-reaching behaviours that involve taking risks, such as skipping security regulations [50]. These behaviours are positively associated with dealing with stressful work situations and achieving goals. They thereby support self-esteem and satisfaction, but they are also associated with deteriorating employee health [50].

In the long term, self-endangering coping behaviour leads to heavy workloads and reduced performance, well-being, and health [52]. Therefore, self-endangering coping behaviour is a vicious circle, as mentioned above. Even if such problems are recognized, employees do not manage to change anything and even behavioural prevention approaches, such as stress management seminars and self-discipline, appear to be insufficient [53].

Whereas Krause et al. (2010) [53] posit that employees exhibit self-endangering coping because of career aspiration, we propose that for nurses, it is based on an altruistic attitude, the desire to gain a boost in self-esteem by helping others, and on team identification.

### 6.2 Altruistic values

Several participants mentioned for example that “you put other people before yourself” and “you do not think of yourself” at work. In addition, when we asked the executives about the nurses’ job motives, all leaders mentioned that it is “love for fellow human beings,” “the desire to help others,” the possibilities to enter into deep relationships, and a “feeling of being loved and needed” that motivates nurses in their job.

In line with these results, previous studies found out that altruism is the most important work value for nurses [[54], [55], [56]]. Whereas Saito et al. (2018) [57] found that altruistic work values have the potential to buffer against nurses’ burnout symptoms, our results indicate that being altruistically motivated in the context of nursing often implies filling in for colleagues more often, working overtime (excessively), or presenteeism. “Yeah, that’s a bit of the helper syndrome. I do think that it is more pronounced in nursing than average. I go into the profession to help, I see the necessity and yes, I know exactly, if I do not step in then my colleague has to do it, or in the end, the residents are perhaps not well cared for and there is already extreme responsibility.” Adjacent to this, recent research has found that, as described above, work intensification can deteriorate employees’ health [50,58]. Based on the observed altruistic attitude, it is likely that the desire to help others is not limited to the process of patient care but also has an impact on the team, such as on team identification.

### 6.3 Team identification

At our workshops, nurses often reported that they sometimes miss appreciation and open communication culture in their team and that colleagues badmouth others behind their backs. Participants also said that “better to save your colleague's weekend than your own” or that they do not want to leave colleagues in the lurch. We therefore conclude that team culture in the elderly care is typically characterized by strong identification with colleagues; however, this can be associated with the expectation that everyone always stands in for colleagues: “Yeah, I would just say they identify with the team, the people who fill in.”

It seems that when nurses fill in for others, they feel responsible for the well-being of the other team members, such that they would rather take on the extra workload themselves than burden their colleagues with it. According to Krause et al. (2010) [53], especially when goals are set at a team level, employees develop the expectation that colleagues will contribute strongly or perform at the same level, and as a result, weaker or sick people are excluded. Additionally, peer pressure increases when no coverage is provided by the company, and therefore one's own limits are increasingly ignored [53].

Although a recent meta-analysis found that organisational and work group identification is positively associated with employees’ health [59], we assume a negative relation based on our results. We suspect that nurses in long-term care who identify with their colleagues are more likely to show self-endangering behaviour, which means filling in for others and coming to work even when ill, which leads to poor mental health in the long term.

### 6.4 Self-worth

Kupcewicz and Józwik (2020) [60] already found out that global self-esteem is a predictor of burnout in nurses in Poland. They conclude that nurses with low self-esteem often experience symptoms of burnout and were more likely to experience personal burnout than work-related burnout [60].

Because many of the nurses in this study reported that they are often not able to delimit themselves from work or special demands or say no to extra work or filling in for colleagues, we further assume that the more nurses have less self-worth the more they define their self-worth through their work; they therefore show more self-endangering behaviour so they can boost their self-esteem.

### 6.5 Theoretical implications of self-endangering in nursing

In summary, recent research shows positive impacts of altruism [57] and team identification [59] on psychological well-being, whereas based on our findings, we suggest negative impacts. However, for less self-care [61,62] and low self-worth [60], previous research found negative influences on mental health, but a more detailed examination in the context of self-endangering has not yet been investigated. Due to these partly contradictory or not yet considered relations, we see great potential in investigating possible mediator effects, which is why we attach particular importance to self-endangering on the basis of our findings.

**Fig. 2:**
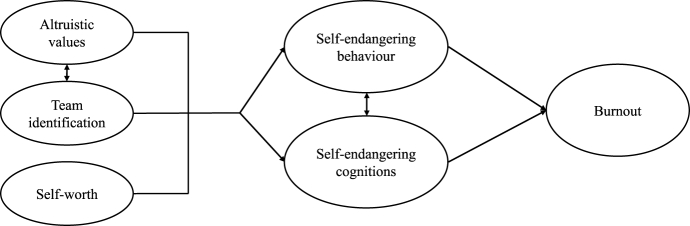
The mediating effect of self-endangering between individuals' ressources and the development of burnout.

We therefore assume that the antecedents altruism and team identification play an important key role in the emergence of self-endangering in nursing, such that high levels of these variables promote self-sacrificing cognitions and behaviour. Low levels of self-worth are a further risk factor for self-endangering, such that low levels of self-worth promote self-sacrificing cognitions and behaviour. In sum, we assume that nurses who often show self-endangering behaviour and cognitions tend to develop burnout symptoms, in the way that nurses self-sacrifice themselves for their work and go beyond their boundaries (see Fig. 2).

Previous research already shows the importance of other constructs that are related to self-endangering behaviour for nurses’ burnout such as work engagement [63], organizational citizenship behaviour [64] and workaholism [65]. By introducing the construct of self-endangering in nursing, we believe that we can broaden the perspective of the previous operationalisations to include the fill-in situation in the event of absenteeism, which is very important for nursing care. Future research can now examine whether the constructs mentioned above predict self-endangering behaviour. Regarding COR theory, self-endangering cognitions might be an important mechanism behind the downward spiral of resource losses, such that it might be particularly difficult to develop a healthy self-care concept for nurses who cannot say no in cases of the need to fill in and feel guilty towards residents and colleagues. Especially with regard to the altruistic personality tendencies and perceived lower self-worth, more quantitative longitudinal research is required.

For future leadership research, self-endangering in nursing implies that the leader’s role in the development of self-endangering behaviour and cognitions has to be examined; protecting vulnerable staff through leadership behaviour seems crucial.

### 6.6 Consequences for further interventions in nursing care

With a view to the important role of self-endangering and the possible antecedents, further interventions in the context of health promotion programmes should focus on nurses’ special job motivation and attitudes to reduce self-sacrificing within their work. It therefore seems extremely important to train nurses in self-reflection and self-empathy, such as the training in the empCARE programme [66], but even more in reflecting upon their desire to help others and their understanding of team work and team identification.

In sum, this means that we need holistic training programmes in nursing care that include: 1) individual training, e.g. coaching, where motivation and personal attitudes toward the job and the definition of one’s own self-esteem are discussed and reflected upon, with the aim that nurses are sensitized and develop personal strategies, 2) team-based interventions, where the team reflects upon their own team values, rules, and culture, with the aim to develop behavioural rules for a healthy cooperation especially in the case of failures, and 3) organisational development processes that reflect upon the organisational structures and culture, with the aim that wards in hospitals and living areas in long-term care facilities are well organised [67].

Finally, we need to stress that identifying self-endangering as a potential individual risk factor for nurses’ burnout does *not* entail that a problematic quality on the side of the nurses is partially responsible for their psychological health issues. Self-endangering only surfaces in the presence of adverse working conditions such as too few staff and too many residents. If working conditions in nursing were not as problematic as they are, nurses would not face the difficult choice between endangering themselves for the sake of their colleagues and residents and taking care of their own health needs.

### 6.7 Limitations

To our knowledge, this study is the first to examine self-endangering behaviour in nursing in long-term care. Of course, our study is not without limitations, especially because we focused on only German nurses in elderly care. Therefore, the first limitation is that our results are not generalizable for nurses in other working contexts or from other cultures. Second, our sample is not representative, so further research is needed in the context of nurses in elderly care to further examine our findings. Nevertheless, we believe that our study offers new knowledge and approaches in the context of the health of elderly care workers.

## 7 Conclusions

This qualitative study dealt with the question of the challenges faced by employees in elderly care and the opportunities for change that arise from their perspective. Our results show that especially the need for filling in for colleagues is one of the greatest challenges for nurses in elderly care. We found different conditioning factors for nurses’ self-endangering at work, such as the value of altruism, strong identification with the team, and low occupational self-care abilities. Future research needs to investigate whether quantitative verification confirms these assumptions.

Adjacent to this, new interventions and programmes can be developed for and implemented in nursing homes to prevent nurses’ burnout and other mental and physical problems. In the long term, this could break the vicious circle of filling in, presenteeism, and illness and enable employees to work more healthily in their everyday work. Of course, it must not be forgotten that these preventive approaches can only be effective in particular or under certain circumstances if working conditions in the nursing sector are also changed.

## Funding

The publication of this article was funded by 10.13039/100009117Chemnitz University of Technology. This research was partially supported by funds from an association of several German public long term-care organisations under the joint project Healthy Ageing in Long-Term Geriatric Care (gesaPflege).

## CRediT authorship contribution statement

**Lara Luisa Eder:** Conceptualization, Methodology, Formal analysis, Investigation, Writing – original draft, Visualization, Project administration. **Bertolt Meyer:** Formal analysis, Resources, Writing – review & editing, Supervision, Funding acquisition.

## Declaration of competing interest

We have no known conflict of interest to disclose.
