# Supplementary material for: Self-endangering: A qualitative study on psychological mechanisms underlying nurses’ burnout in long-term care
Source: Int J Nurs Sci. 2021 Dec 15;9(1):36–48. doi: 10.1016/j.ijnss.2021.12.001 (PMC8766778; doi:10.1016/j.ijnss.2021.12.001)
Supplement: Multimedia component 1 [file mmc1.docx]

自我损耗：长期照护机构中护士职业倦怠的心理机制研究

Lara Luisa Eder, Bertolt Meyer

【摘要】

目的 了解长期照护机构护士发生职业倦怠的心理机制，为制订改善护士心理健康的干预策略提供基础。

方法 采用主题分析法进行两项定性研究。第1项研究于2019年5—7月在德国8家养老院以工作坊形式对110名护士进行了8次小组访谈。作为补充，第2项研究于2019年12月对德国养老院的14名管理人员进行半结构访谈。

结果 第1项研究确定的3个主要主题包括：挑战的原因、员工的变革机会和组织的变革机会。第2项研究确定的3个主要主题包括：工作动机、替班原因和员工的自我照顾。研究还发现，在应对挑战的过程中，自我损耗——当被要求替班或加班时说不的能力减弱——是护士职业倦怠的重要前因。此外，人力资源不足时员工高水平的利他动机和团队或组织认同感与自我损耗有关；自我价值感低也是导致自我损耗的风险因素。

结论 关于工作需求与职业倦怠的经典理论模型中常将动机和认同作为资源，但本研究发现与经典理论模型中的一些核心原则不一致。应制订整体性护理干预计划，包括个人辅导、团队干预和组织发展程序。员工自身在长期照护工作中对这一问题要敏感，并应得到支持；政策制定者应该建立合理的资源结构，即不再鼓励这种员工自我损耗行为的结构。

【关键词】利他主义；医疗护理提供者；卫生人员；精神卫生；医护疗养所；职业倦怠；社会认同；工作负荷量

通信作者：Lara Luisa Eder, E-mail: lara-luisa.eder@psychologie.tu-chemnitz.de
