# Supplementary material for: Self-endangering: A qualitative study on psychological mechanisms underlying nurses’ burnout in long-term care
Source: Int J Nurs Sci. 2021 Dec 15;9(1):36–48. doi: 10.1016/j.ijnss.2021.12.001 (PMC8766778; doi:10.1016/j.ijnss.2021.12.001)
Supplement: Multimedia component 2 [file mmc2.docx]

**Appendices**

**Appendix A. Residential eldercare system in Germany**

**Appendix B. Detailed description of the contextual factors and the sample**

**Appendix C. Supplemental information about the workshops and the detailed schedule for data collection of Study 1**

**Appendix D. Interview questions for data collection of Study 2**

The supplemental file can also be found online (anonymous link for peer review: https://osf.io/72ptu/?view_only=4684b88782a1447f9888762ba39536a9)

**Appendix A**

**Residential eldercare system in Germany**

There is a major shortage of skilled workers in the care sector throughout Germany (Bundesagentur für Arbeit, 2020). This shortage reflected in an insufficient number of applicants, in difficulties with filling vacancies promptly, and in the declining levels of qualifications of applications (Isfort et al., 2018). The reasons for the low attractiveness of the profession of elderly care include physically and mentally challenging working conditions, shift work, low pay, and the allegedly low reputation of the profession. This shortage is one of the reasons for the increasing numbers of sick days, and the increasing amount of overtime among working nurses in Germany (Isfort et al., 2018).

It is therefore not surprising that the average length of stay of a certified nurse in nursing homes in their profession is only 8.4 years (Techniker Krankenkasse, 2017) and that politicians and funding agencies are not succeeding in binding the qualified personnel to their profession in the long term with good working conditions. These problems seem to be widespread in post-industrial countries in general (O’Brien-Pallas et al., 2001; Sochalski, 2001), resulting in increasing dissatisfaction and turnover intentions among nurses (Gasparino, 2014; Leiter & Maslach, 2009). In summary these developments highlight the necessity for better understanding how working conditions stress nurses.

**Appendix B**

**Detailed description of the contextual factors and the sample**

**--General Information for Study 1 and Study 2**

This study is part of a long-term research project in North Rhine Westphalia in Germany which aims to initiate an organisational development process in order to improve the employees’ and residents’ health. The three-year project started in 2017 and will culminate in an action guide for care facilities at the end of the project.

All participating nursing homes were located in North Rhine Westphalia in Germany. The sponsorship, location and size of the nursing homes were mixed. All nursing homes were specialized in elderly long-term care. The number of employees varied between 50 and 130.

The project's structure was oriented towards a typically PDCA cycle (Schmitt & Pfeifer, 2015) and as part of the needs analysis of this project, we conducted an employee survey and supplemental workshops with employees.

The scales covered in the employee survey were:

- Work climate (COPSOQ – Nübling, 2005)
- Workplace bullying (COPSOQ – Nübling, 2005)
- Social support colleagues & supervisors (COPSOQ – Nübling, 2005)
- Burnout (CBI-Skala – Kristensen et al., 2007)
- Qualitative workload (BGW), 2013)
- Job satisfaction (COPSOQ – Nübling, 2005)
- Health orientated leadership (Franke & Felfe, 2016)
- Health awareness and behaviour (Franke & Felfe, 2016)
- Identification with the team (Hogg & Terry, 2000)
- Communication (Pfaff et al., 2004)
- Team Performance (Peirce & Sims, 2002)
- Presenteeism (own conception)
- Work ability (Work Ability Index – Tuomi et al., 1994)
- Health state (EQ-5D – Herdman et al., 2011)
- Emotion work (Zapf et al., 2000)
- Work situation (own conception)
- Cooperation (Zill et al., 2018 ; Braun, 2015)
- Leadership quality (COPSOQ – Nübling, 2005)
- Appreciation (Eisenberger et al., 1997)
- Work-Privacy conflict (Netemeyer et al., 1996)
- Mourning (own conception)
- Information flow (own conception)
- Health and prevention courses (own conception)
- Perception of courses (own conception)
- Stress due to relatives (own conception)

The workshops aimed to generate additional content and, above all, identify causes for challenges. The process was interventionist in order to identify the different needs in advance. The first author conducted the workshops (Study 1) and the interviews (Study 2). The first author is female, a psychologist (Master of Science) and experienced in dealing with consulting contexts and workshop implementation. The first author knew the selected Nursing Homes from her involvement in the research project so she was familiar with the structures, data on the health of employees in the nursing home, and the employees and leaders. The first researcher carried out the employee workshops following the employees’ survey results, which has been part of the previous analysis steps in the health care project. The first author’s prior knowledge of the organisations helped insight of the aim of this study to provide a deeper understanding of the qualitative results and their relations. In addition, all project institutions were informed that the first author was conducting various studies for her PHD as part of the project.

**Appendix C**

**Supplemental information about the workshops and the detailed schedule for data collection of Study 1**

The workshop was organised in the style of a so-called World Café, i.e. each of the five topics was assigned to a table. Participants were split into five groups and rotated across the tables. Participants spent 10 to 15 minutes at each table, discussing the topic, before rotating to the next. Discussions at each table were structured by five guiding questions that we developed. These questions were part of psychological organisational development process in the health care project mentioned above based on a classic planning-doing-checking-acting (PDCA) cycle (Schmitt & Pfeifer, 2015) and using our previous knowledge of the nursing homes in cooperation with our external project partners in the health care project, who are experts in workplace health promotion and are experienced in workshop implementation. The five questions were:

1. *What are the causes of XX* [e.g. high burnout symptoms, high rates of presenteeism]*?*
2. *How does that make me feel? (for example, anxious, ill, aggressive)*
3. *What must happen for the condition to improve?*
4. *What can I contribute to that?*
5. *What can the institution contribute to that?*

After each group had finished with all topics, we discussed the results in the large group and added supplements if necessary up to the point of data saturation.

**Appendix D**

**Interview questions for data collection of Study 2**

We used this existing knowledge about the reasons for the challenges and the possible aspects of change to develop these interview questions:

1. *What would you say drives nursing staff in particular to exercise their profession? What professional motives could play a role here in your view?*
2. *How is the provision of nursing care in your home regulated in the event of illness or absences? How do you, as a manager, deal with the situation when a loss has to be compensated/replacement has to be found? Are there people who fill in particularly often and/or are there people who rarely or never do so? What distinguishes the people who fill infrequently from those who never do?*
3. *What role does the topic of self-care and health behaviour play here, for example, "doing something good" for oneself at work? (e.g. participating in workplace training courses; allowing yourself a short break, "just going out for a moment"; taking advantage of opportunities for compensation outside work)?*
4. *Do you have the feeling that different professional groups differ on average in how well they can differentiate themselves?*
